# The lasting effects of childhood trauma on developing psychiatric symptoms: A population-based, large-scale comparison study

**DOI:** 10.1017/gmh.2024.100

**Published:** 2024-10-24

**Authors:** Yu Jin, Shicun Xu, Zhixian Shao, Xianyu Luo, Amanda Wilson, Jiaqi Li, Yuanyuan Wang

**Affiliations:** 1 Department of Statistics, Faculty of Arts and Sciences, Beijing Normal University, Beijing, China; 2 Northeast Asian Research Center, Jilin University, Changchun, China; 3 Faculty of Health and Life Sciences, De Montfort University, Leicester, UK; 4 Key Laboratory of Brain, Cognition and Education Sciences, Ministry of Education, China; School of Psychology, Center for Studies of Psychological Application, and Guangdong Key Laboratory of Mental Health and Cognitive Science, South China Normal University, Guangzhou, China

**Keywords:** childhood trauma, propensity score matching, hierarchical regression, psychiatric symptoms

## Abstract

**Background:**

Childhood trauma (CT) increases rates of psychiatric disorders and symptoms, however, the lasting effect of CT into adulthood has little exploration using large-scale samples.

**Objectives:**

This study estimated the prevalence of CT in a large sample of Chinese young adults, examining the risk factors of current psychological symptoms among those with CT experiences.

**Methods:**

117,769 college students were divided into CT and non-CT groups. The propensity score matching method balanced the confounding sociodemographic factors between the two groups, compared to 16 self-reported psychiatric disorders (e.g., depression, anxiety, eating disorder, obsessive-compulsive disorder, autism, social anxiety disorder, post-traumatic stress disorder), and seven current psychiatric symptoms. Hierarchical regression employed the significant risk factors of the seven current psychiatric symptoms.

**Results:**

The prevalence of CT among young adults was 28.76% (95% CI: 28.47–29.04%). Youths with CT experiences reported higher psychiatric disorder rates and current symptom scores (P < 0.001). Sociodemographic factors (females, family disharmony, low socioeconomic status, poor relationship with parents, lower father’s education level) and lifestyle factors (smoking status, alcohol consumption, lack of exercise) were significantly associated with current psychiatric symptoms.

**Results:**

Public health departments and colleges should develop strategies to promote mental health among those who have experienced CT.

## Graphical abstract

**Figure:**
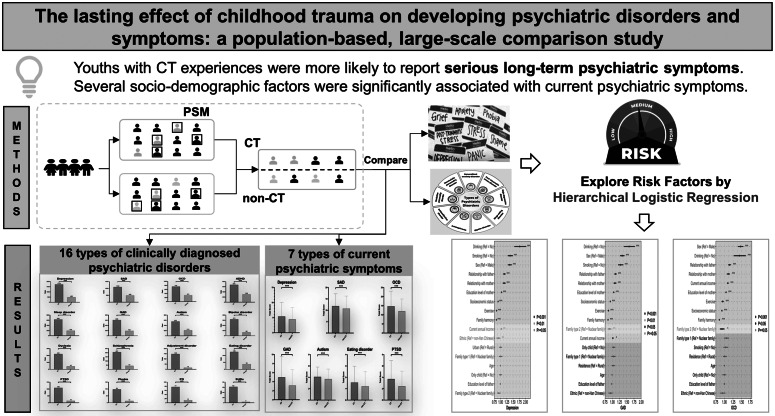

## Impact statement

This large population-based, cross-sectional study conducted in China offers critical insights into the correlation between childhood trauma (CT) and the self-reported prevalence of psychiatric disorders among young adults. It reveals that individuals with a history of CT are at a heightened risk of experiencing serious and persistent psychiatric disorders and symptoms, necessitating a targeted approach to mental health support. The research also emphasizes the role of demographic factors as potential risk indicators, suggesting the importance of considering these variables when designing interventions. The implications of these findings extend to the need for public health and academic sectors to collaboratively develop strategies aimed at improving mental health outcomes and overall quality of life for youth, with a particular focus on those who have experienced CT. Additionally, the study underscores the importance of an interdisciplinary approach, and the potential for future longitudinal research to further our understanding of the long-term effects of CT on mental health. This underscores the urgency for policy-makers and healthcare providers to prioritize and invest in comprehensive mental health services, especially for high-risk groups.

## Introduction

Childhood trauma (CT), or childhood maltreatment refers to all forms of emotional and physical mistreatment, sexual abuse (SA), neglect and other traumatic experiences during childhood (World Health Organization, 2014), and has been internationally recognized as a serious and urgent public health problem. Moreover, exposure to CT has profound and lasting effects on an individual’s mental health and well-being, in terms of depression, anxiety, post-traumatic stress disorder (PTSD), personality disorders, substance use disorders (SUDs), sexually transmitted infections (STIs) and even suicidal behaviors (Zatti et al., 2017; McKay et al., 2021; Bauer et al., 2022). Thus, it is vital to investigate the lasting effects of CT and related risk factors, and apply early screening and intervention for young adults with CT experiences.

CT and childhood maltreatment raise significant mental health concerns on an international level. Specific prevalence rates can vary widely between countries and regions due to differences in culture, socioeconomic conditions and the availability and quality of data. Several systematic reviews and meta-analyses (Massullo et al., 2023) suggest the prevalence of CT among young adults’ ranges from 13.4% to 64.7% depending on the country, such as 13.4% in Germany, 34.3% in Brazil, and 64.7% in China (Witt et al., 2017; Fu et al., 2018; Bauer et al., 2022). There is a consistently high prevalence of child maltreatment in the East Asia and Pacific regions. Between one in 10 children experience physical abuse (PA) and 30.3% of children suffer from abuse according to a systematic review of data from the East Asia and Pacific regions (Fry et al., 2012). According to another report, the individual prevalence rates for PA, emotional abuse (EA), SA and neglect in China are 26.6%, 19.6%, 8.7% and 26.0%, respectively (Fang et al., 2015). It is important to note that these figures are estimated based on outdated data and that the actual prevalence may be higher, as well as due to other common factors such as underreporting. Regardless, these high rates highlight the extent of CT and underscore the urgent need for interventions and support systems to address this issue, particularly in China.

Exposure to CT has been shown to cause deleterious physical and psychological outcomes that can persist into adulthood (Bauer et al., 2022). Several systematic reviews and meta-analyses have reported that young adults exposed to any form of CT are at an increased risk of chronic psychological disorders (Read et al., 2005; Wu et al., 2010; Norman et al., 2012; Hughes et al., 2017; McKay et al., 2021; Park et al., 2021). For example, a meta-analysis using a longitudinal cohort study found that experiencing CT results in more than three times the odds of developing a psychiatric disorder (OR = 3.11, 95% CI: 1.36–7.14 McKay et al., 2021). Another cohort cross sectional and case controlled meta-analysis found significant associations between CT and depressive disorders, suicide attempts, SUDs and STIs (Norman et al., 2012). Furthermore, several studies also reported that exposure to CT could increase the risk of depression, anxiety, PTSD, psychosis, SUDs, attachment disorder and suicidal behaviors (Read et al., 2005; Wu et al., 2010; Hughes et al., 2017; Park et al., 2021; Bauer et al., 2022). Neurobiology studies of CT further suggested experiences of CT would cause long-term neurobiological changes that impact individual development brain function (Hesdorffer et al., 2009), such as brain circuits, hormonal systems and the hypothalamic–pituitary–adrenal (HPA) axis, which affects the ability to modulate behavioral and cognitive responses to subsequent stress (Nemeroff, 2004; Assogna et al., 2020). Considering the serious mental health consequences of CT, it is crucial to investigate the risk factors of youth who have had CT experiences to implement effective measures to improve their quality of life and well-being.

Several researches have explored the risk factors (i.e., psychosocial, environmental and genetic) of psychiatric disorders among CT survivors. Systematic reviews reported that sex, race, ethnicity, educational level, lower social status could be moderators for CT and psychopathology (Jaffee and Maikovich-Fong, 2011; Petruccelli et al., 2019; Kisely et al., 2020). Social support, self-esteem, self-reliance and lifestyle factor are associated with psychiatric disorders among those who have experienced CT (Horan and Widom, 2015; Xiao et al., 2023). For instance, females report more CT than males and are more likely to have negative health outcomes. A non-white race/ethnicity, lower educational level and lower socioeconomic status have all been significantly associated with CT experiences (Petruccelli et al., 2019). Furthermore, tobacco use and increased alcohol consumption also have shown associations with CT in both adjusted and unadjusted models (Petruccelli et al., 2019; Xiao et al., 2023).

However, few studies utilized a large sample size and few studies control for confounding factors (i.e., some sociodemographic factors) when exploring the effects of CT among young adults, especially the combination of psychiatric disorders and current psychiatric symptoms. Therefore, this study conducted a large-scale survey covering more than 110,000 Chinese youth to investigate the long-term psychological consequences of CT as well as risk factors after controlling for confounding sociodemographic factors. The aims of this study were 1) to estimate the prevalence of CT among Chinese young adults; 2) to investigate 16 self-reported psychiatric disorders in CT youth compared with non-CT youth after controlling for confounding factors; and 3) to examine the risk factors for seven types of psychiatric symptoms among youth with CT experiences. The hypotheses of this study were 1) after controlling for confounding factors, there would be significant differences of prevalence and severity of psychiatric disorders between CT and non-CT groups; 2) several sociodemographic factors, such as age, sex, residence and current annual family income, are expected to demonstrate significant associations with an elevated risk of current psychiatric symptoms among youth with CT experiences.

## Methods

### Study design and settings

This large-scale cross-sectional study was undertaken by Jilin University, China, from October to November 2021, covering 63 colleges and universities in Jilin province. The study design followed the Strengthening the Reporting of Observational Studies in Epidemiology (STROBE) reporting guideline (Von Elm et al., 2007), a convenience sampling method was used in this study. The quick response code (QR code) was linked to the web-based self-administered questionnaire and this was distributed to participants online via on the official accounts of each college and university. The inclusion criteria were: 1) currently enrolled in colleges and universities in Jilin province; 2) aged 15 years and older; 3) possess a satisfactory comprehension of the assessment content and the simplified Chinese language. This study received ethical approval from Jilin University (N020210929 [11 October 2021]) in accordance with the principles of the 1964 Helsinki Declaration and its 2013 amendments (World Medical Association, 2013). Electronic informed consent was obtained from all participants.

**Figure 1.**
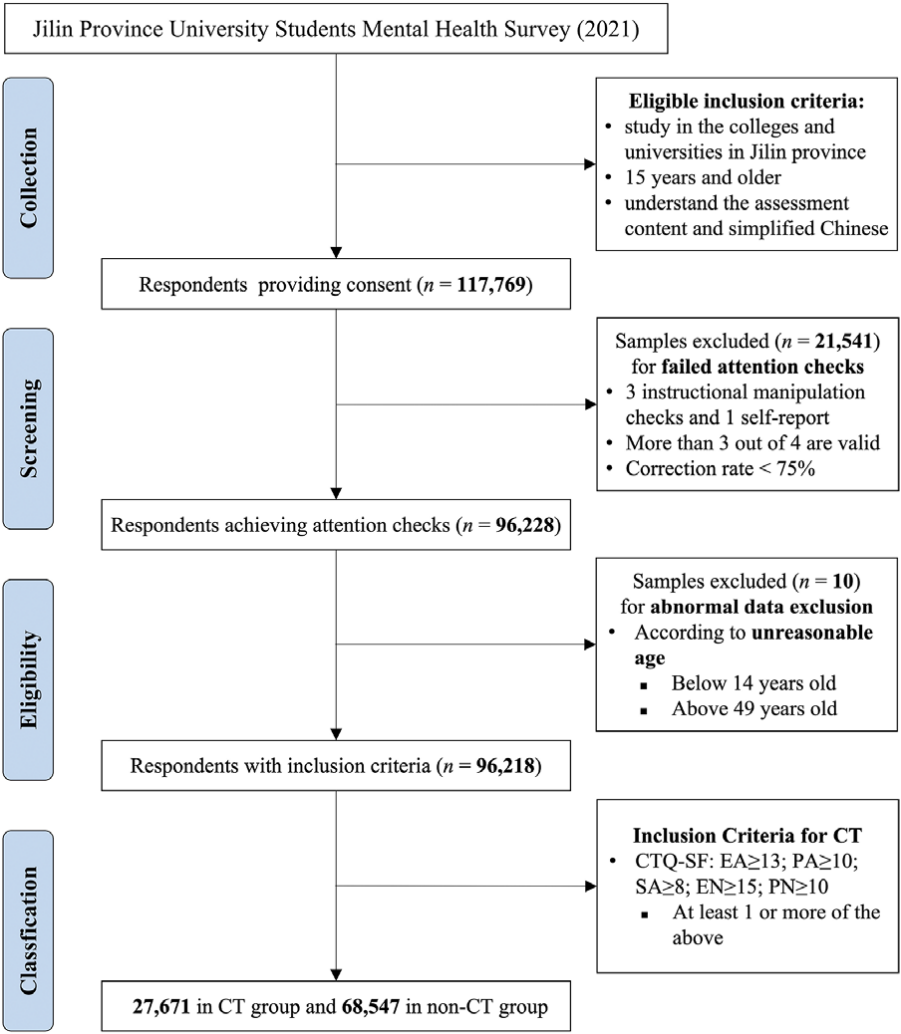
Flowchart of recruitment procedures. *Abbreviations*: CT, childhood trauma; CTQ-SF, Childhood Trauma Questionnaire-Short Form; EA, emotional abuse; EN, emotional neglect; PA, physical abuse; PN, physical neglect; SA, sexual abuse.

A total of 117,769 students participated and completed the questionnaire in this survey during the data collection period. Figure 1 provides an overview of the participant screening and exclusion process. Out of the 117,769 individuals, a total of 21,551 participants were excluded, including those who failed attention checks (21,541) and 10 with suspected abnormal data according to unreasonable age, height and weight values. This resulted in a final sample of 96,218 participants, representing a response rate of approximately 81.7%. In terms of questionnaire design, the relevant assessment scales were administered and the basic sociodemographic characteristics were collected, including age, sex at birth, residence, current annual family income, socioeconomic status, only child status, ethnicity, smoking status, consuming alcohol and exercise.

### Measurements

#### Childhood trauma

CT experiences were measured using the Chinese version of the Childhood Trauma Questionnaire-Short Form (CTQ-SF), a self-report inventory consisting of 28 items rated on a five-point Likert-type scale ranging from 1 (never true) to 5 (very often true; Bernstein et al., 2003). The CTQ-SF is designed to assess five categories of CT, EA, emotional neglect (EN), PA, physical neglect (PN) and SA, identified by moderate to severe cutoff scores in each subscale. Specifically, cutoff scores of 13 or higher for EA, 15 or higher for EN, 10 or higher for PA, 10 or higher for PN and 8 or higher for SA (Alexander et al., 2018). Individuals with at least one type of abuse identified were divided into the CT group. The modified Chinese version of CTQ-SF has demonstrated excellent reliability and validity, with a Cronbach’s alpha of 0.79 among Chinese participants (He et al., 2019).

#### Assessment of 16 psychiatric disorders

The 16 psychiatric disorders were to be diagnosed by psychiatrists, however, the psychiatric disorder was self-reported by the participants. Participants were asked the question, “Have you ever been diagnosed by a psychiatrist with any of the following psychiatric disorders? (You can select multiple).” With choices including: (1) autism; (2) attention-deficit hyperactivity-disorder (ADHD); (3) depression; (4) bipolar disorder; (5) generalized anxiety disorder (GAD); (6) obsessive–compulsive disorder (OCD); (7) schizophrenia; (8) phobia; (9) PTSD; (10) panic disorder (PD); (11) SUDs; (12) learning disabilities/dyslexia; (13) sleep disorders; (14) adjustment disorders; (15) eating disorders; (16) social anxiety disorder (SAD); (17) other (fill in the blank); (18) none of the above.

#### Assessment of seven types of current psychiatric symptoms

The seven current psychiatric symptoms included: depression, GAD, eating disorders, OCD, autism, SAD and PTSD, and were measured by their respective self-report scales. The Patient Health Questionnaire-9 (PHQ-9), has nine items, assessing depressive symptoms over the last 2 weeks using a cutoff value of 5 (Kroenke et al., 2001). A higher score on this scale indicates a higher level of depressive symptoms. Participants choose the questions that have bothered them over the past 2 weeks, with responses ranging from one (not at all) to three (nearly every day). It has demonstrated good performance in the Chinese population, with a sensitivity of 86% and a specificity of 86% (Wang et al., 2014) The Generalized Anxiety Disorder (GAD-7) scale assesses anxiety (Spitzer et al., 2006), with a cutoff point of 5 to classify respondents as having high (5 or higher) or low (less than 5) levels. This scale has demonstrated excellent sensitivity and specificity (>85%) in its application within China (He et al., 2010). The Sick Control One Fat Food (SCOFF) questionnaire measures eating disorders, and a score of 2 or higher indicates a likely positive case (Morgan et al., 1999). The scale exhibits a sensitivity of 97.7% and a specificity of 94.4% (Kutz et al., 2020). The Dimensional Obsessive–Compulsive Scale-short Form (DOCS-SF) evaluates OCD providing a brief (five-item) measure of OCD symptoms and has a suggested cutoff score of 16 to diagnose negative or positive behaviors, while demonstrating a sensitivity of 96% and a specificity of 94% (Eilertsen et al., 2017). The 10-item Autism Spectrum Quotient (AQ-10) assesses autism symptoms through a specialist evaluation. A cutoff point greater than or equal to 6 indicates the presence of autism symptoms (Allison et al., 2012), and it has a sensitivity of 74% and a specificity of 85% (Leung et al., 2023). The subscale of self-consciousness measured SAD, rates higher scores as associated with worse symptoms (Fenigstein et al., 1975). The Chinese version of the subscale of self-consciousness has a reliable internal consistency, with a Cronbach’s alpha of 0.79 (Shek, 1994). The Trauma Screening Questionnaire (TSQ, Chinese version) identifies the severity of potential PTSD, adapted from the PTSD Symptom Scale – Self-Report Version (Foa et al., 1993), which has been utilized in various samples across countries (Bernstein et al., 2003; Walters et al., 2007; Wu, 2014; Knipscheer et al., 2020). It consists of five re-experiencing items (e.g., “upsetting dreams about the event”) and five arousal items (e.g., “difficulty falling or staying asleep”). Participants are asked to answer the question of whether they had experienced these items before, using “Yes” (scored 1) or “No” (scored 0). Six or more positive responses indicated that the respondent was at risk of PTSD. It shows good internal consistency (Cronbach’s alpha coefficient of 0.93) in Chinese university students (Wu et al., 2019).

#### Collection of sociodemographic variables

Our study collected sociodemographic variables through self-report measures, including current annual family income and socioeconomic status. To assess the variable “current annual family income,” participants were asked the following question: “What is your household’s current annual income?” This question sought to determine the average amount available for expenditure and savings per person in their household. To assess the variable “socioeconomic status,” participants provided self-reported data, rating their perceived socioeconomic status using the Chinese version of the MacArthur Scale of Subjective Socioeconomic Status (Adler et al., 2000; Xiaona and Xiaoping, 2018). This scale involves participants selecting a number from 0 to 10, using a ladder figure, with higher numbers indicating a higher perceived socioeconomic status.

### Statistical analysis

#### Propensity score matching

The propensity score matching (PSM) method was utilized to balance the potential confounding factors between the CT and non-CT groups. Propensity scores were calculated by a logistic regression model, minimizing the influence caused by a set of unmatched sociodemographic characteristics, including sex, age, ethnicity, residence, only-child (yes or no), current annual family income and socioeconomic status. Based on the propensity scores, participants were paired 1:1 using the nearest neighbor method, with a caliper width equal to 0.2 of the standard deviation of the logit of the propensity score (Austin, 2011). In addition, the standardized mean difference (SMD) was computed to assess balance after PSM, where a SMD less than 0.1 indicated a substantial balance (Zhang et al., 2019). Furthermore, the independent sample *t*-test and chi-squared test were performed before and after the PSM procedure, using the aforementioned sociodemographic characteristics among the two groups, CT and non-CT. The PSM procedure was conducted with the “MatchIt” package in R. After balancing the confounding factors, the prevalences of self-reported psychiatric disorders were compared with the chi-squared test between the CT and non-CT groups, while the total score of current psychiatric symptoms were compared with the independent sample *t*-test between the two groups. All analysis procedures were performed with R software, with the significance level (α) preset at 0.05 for all two-tailed tests.

#### Hierarchical regression model

In order to identify risk factors of current psychiatric symptoms from sociodemographic characteristics and family-related factors in the youth with CT experiences, a hierarchical regression procedure was conducted by assigning the above covariates to different blocks. The hierarchical logistic regression method was selected for the six types of psychiatric symptoms, which corresponds with the scales used in this study that had definite cutoff values, and the psychiatric symptoms were taken as the response variable. For SAD, without a clear cutoff value, the hierarchical linear regression method was used to explore the risk factors. Both the hierarchical logistic regression and the hierarchical linear regression were performed with SPSS version 26. All null hypothesis significance testing was conducted at the two-tailed level with significance of 0.05.

## Results

### Control for confounding factors

Following the application of the inclusion and exclusion criteria, 96,218 participants were enrolled, including 68,547 in non-CT group and 27,671 in CT group. The PSM procedure was then performed to balance the distribution of all baseline covariates (i.e., age, sex, residence, current annual income, socioeconomic status, only-child status, ethnicity) between the CT and non-CT groups, ensuring the accuracy and robustness of subsequent analysis results. Summary statistics for the baseline characteristics of CT and non-CT groups before and after PSM are shown in Table 1. After matching, SMDs for all characteristics were <0.10, indicating no significant difference between these two groups. After balancing confounding factors, the CT and non-CT groups comprised 27,671 samples, respectively.

**Table 1.** Baseline characteristics of CT and non-CT groups, before and after PSM (*N* total = 96,218)

	Before PSM (*n* = 96,218)				After PSM (*n* = 55,342)			
	Non-CT	CT			Non-CT	CT		
	(*n* = 68,547)	(*n* = 27,671)	*P* ^1^	SMD	(*n* = 27,671)	(*n* = 27,671)	*P* ^2^	SMD
Age								
Mean (SD)	19.59 (1.74)	19.59 (1.76)	0.69	0.003	19.58 (1.70)	19.59 (1.76)	0.68	0.004
Sex at birth			<0.001	0.22			0.12	0.013
Male	26,360 (38.5)	13,705 (49.5)			13,889 (50.2)	13,705 (49.5)		
Female	42,187 (61.5)	13,966 (50.5)			13,782 (49.8)	13,966 (50.5)		
Residence			<0.001	0.05			0.10	0.002
Urban	35,336 (51.6)	13,596 (49.1)			13,626 (49.2)	13,596 (49.1)		
Suburban/rural	33,211 (48.4)	14,075 (50.9)			14,045 (50.8)	14,075 (50.9)		
Current annual family income			<0.001	0.11			0.65	0.008
<¥14,000	41,616 (60.7)	18,220 (65.8)			18,154 (65.6)	18,220 (65.8)		
¥14,000–35,999	18,938 (27.6)	6,537 (23.6)			6,628 (24.0)	6,537 (23.6)		
≥¥36,000	7,993 (11.7)	2,914 (10.5)			2,889 (10.4)	2,914 (10.5)		
Socioeconomic status			<0.001	0.16			0.35	0.008
Low	51,740 (75.5)	22,641 (81.8)			22,726 (82.1)	22,641 (81.8)		
High	16,807 (24.5)	5,030 (18.2)			4,945 (17.9)	5,030 (18.2)		
Only child status			<0.001	0.05			0.73	0.003
Only child	33,064 (48.2)	12,596 (45.5)			12,637 (45.7)	12,596 (45.5)		
Have siblings	35,483 (51.8)	15,075 (54.5)			15,034 (54.3)	15,075 (54.5)		
Ethnicity			0.02	0.02			0.47	0.006
Han	61,451 (89.6)	24,660 (89.1)			24,714 (89.3)	24,660 (89.1)		
Others	7,096 (10.4)	3,011 (10.9)			2,957 (10.7)	3,011 (10.9)		

*Note*: The numbers in parentheses denote “% of the sample for the corresponding subpopulation (CT and non-CT).” *P* ^1^ represents *P*-value of *t*-test or chi-squared test comparing non-CT to CT samples before PSM. *P* ^2^ represents *P*-value of *t*-test or chi-squared test comparing non-CT to CT samples after PSM.

*Abbreviations*: CT, childhood trauma; PSM, propensity score matching; SD, standard deviation; SMD, standardized mean difference.

### Comparison of self-reported psychiatric disorders

**Figure 2.**
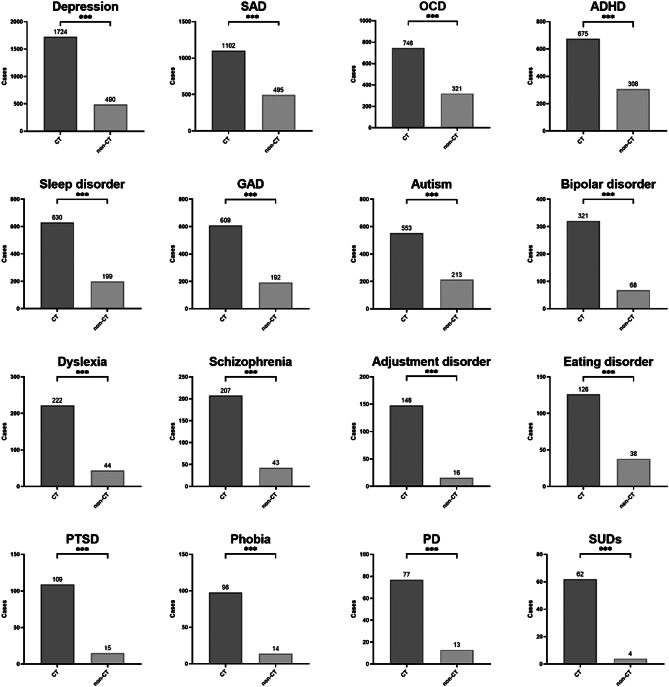
Comparison the number of cases with 16 types of clinically diagnosed psychiatric disorders between the CT and non-CT groups. *Abbreviations*: ADHD, attention deficit and hyperactivity disorder; CT, childhood trauma; GAD, generalized anxiety disorder; OCD, obsessive–compulsive disorder; PD, panic disorder; PTSD, post-traumatic stress disorder; SAD, social anxiety disorder; SUDs, substance use disorders. *P < 0.05, **P < 0.01, ***P < 0.001.

As reported in Table 2, there was a significant higher prevalence of the 16 types of self-reported psychiatric disorders in youth with CT experiences than those without CT experiences (Table 2; *P* < 0.001). Figure 2 demonstrates the differences of participants with the 16 types of self-reported psychiatric disorders between the CT and non-CT groups.

**Table 2.** Differences between CT and non-CT groups in self-reported psychiatric disorders

	Total (*n* = 55,342)		CT (*n* = 27,671)		Non-CT (*n* = 27,671)			
	*n*	%	*n*	%	*n*	%	χ^2^	*P*-value
Depression	2,218	4.0	1,724	6.2	494	1.8	709.42	<0.001
SAD	1,583	2.9	1,102	4.0	481	1.7	249.98	<0.001
OCD	1,073	1.9	746	2.7	327	1.2	166.06	<0.001
ADHD	989	1.8	675	2.4	314	1.1	133.43	<0.001
Sleep disorder	830	1.5	630	2.3	200	0.7	225.11	<0.001
GAD	806	1.5	609	2.2	197	0.7	212.68	<0.001
Autism	765	1.4	553	2.0	212	0.8	153.23	<0.001
Bipolar disorder	391	0.7	321	1.2	70	0.3	160.98	<0.001
Dyslexia	264	0.5	222	0.8	42	0.2	121.95	<0.001
Schizophrenia	250	0.5	207	0.7	43	0.2	106.76	<0.001
Adjustment disorder	163	0.3	148	0.5	15	0.1	107.21	<0.001
Eating disorder	161	0.3	126	0.5	35	0.1	50.46	<0.001
PTSD	126	0.2	109	0.4	17	0.1	65.88	<0.001
Phobia	112	0.2	98	0.4	14	0.1	61.63	<0.001
PD	90	0.2	77	0.3	13	<0.1	44.17	<0.001
SUDs	66	0.1	62	0.2	4	<0.1	49.27	<0.001

*Note*: χ^2^ tests for between-category differences.

*Abbreviations*: ADHD, attention deficit and hyperactivity disorder; CT, childhood trauma; GAD, generalized anxiety disorder; OCD, obsessive–compulsive disorder; PD, panic disorder; PTSD, post-traumatic stress disorder; SAD, social anxiety disorder; SUDs, substance use disorders.

### Comparison of current psychiatric symptoms

**Figure 3.**
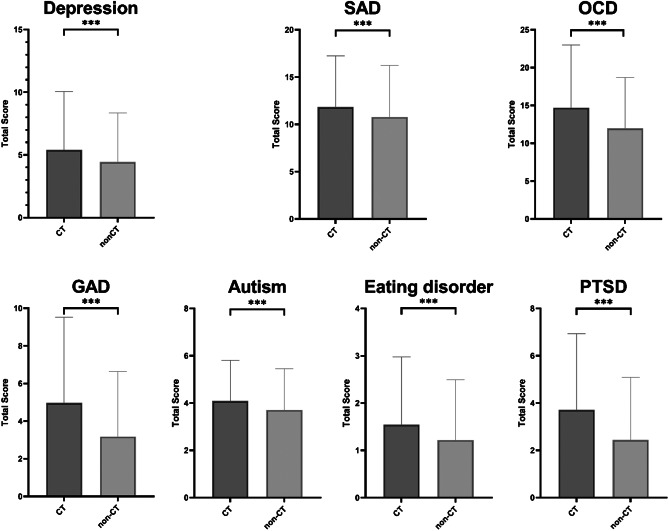
Comparison of total scores of seven current psychiatric symptoms in the CT and non-CT groups. *Abbreviations*: CT, childhood trauma; GAD, generalized anxiety disorder; OCD, obsessive–compulsive disorder; PTSD, post-traumatic stress disorder; SAD, social anxiety disorder.

Table 3 shows that the CT group reported a significantly worse current symptom status regarding seven types of psychiatric symptoms than the non-CT groups (*P* < 0.001). Figure 3 depicts the comparison of total scores in the seven types of current psychiatric symptoms between the CT and non-CT groups.

**Table 3.** Differences between CT and non-CT groups and current psychiatric symptoms

	CT	Non-CT	*t*-test	*P*-value
Depression	6.83 (5.23)	4.54 (4.01)	57.941	<0.001
SAD	11.83 (5.41)	10.66 (5.48)	25.335	<0.001
OCD	14.69 (8.31)	11.87 (6.76)	43.888	<0.001
GAD	4.97 (4.55)	3.19 (3.50)	51.462	<0.001
Autism	4.09 (1.72)	3.72 (1.74)	24.860	<0.001
Eating disorder	1.54 (1.43)	1.20 (1.27)	29.613	<0.001
PTSD	3.71 (3.21)	2.49 (2.66)	48.891	<0.001

*Note*: The numbers out and in parentheses, respectively, denote “mean and standard deviation of the sample for the corresponding subpopulation (CT and non-CT).” Mean and standard deviation are provided for the scores on scales of current psychiatric symptoms above.

*Abbreviations*: CT, childhood trauma; GAD, generalized anxiety disorder; OCD, obsessive–compulsive disorder; PTSD, post-traumatic stress disorder; SAD, social anxiety disorder.

### Risk factors of current psychiatric symptoms

**Figure 4.**
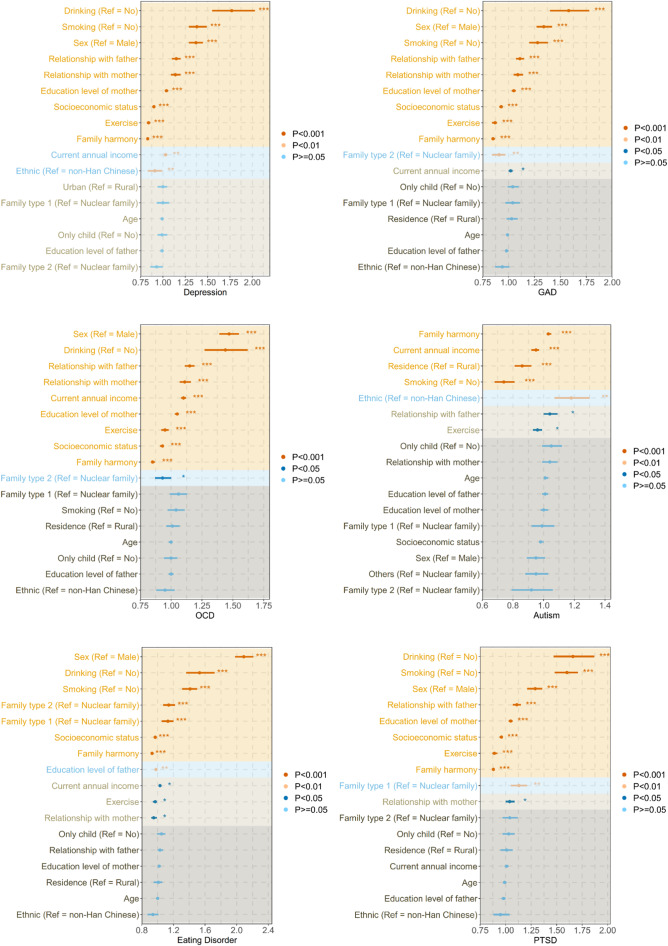
Forest plots for the results of hierarchical logistic regression. *Abbreviations*: GAD, generalized anxiety disorder; OCD, obsessive–compulsive disorder; PTSD post-traumatic stress disorder; Family type 1: More than three generation; Family type 2: Others.

Table 4 presents the summary results of all hierarchical regression models, and total results are presented in Table S1 in the Supplementary Material. The overall outcomes of forest plot are presented in Figure 4.

**Table 4.** Exploring risk factors for current psychiatric symptoms by hierarchical regression analysis among the CT group

Independent variable	Depression	GAD	OCD	Autism	Eating disorder	PTSD	SAD
	OR (95% CI)	OR (95% CI)	OR (95% CI)	OR (95% CI)	OR (95% CI)	OR (95% CI)	*b* (SE)
*Sociodemographic characteristics*							
Age	0.99 (0.98, 1.01)	0.99 (0.98, 1.00)	1.00 (0.98, 1.01)	1.02 (1.00, 1.03)	1.00 (0.99, 1.02)	0.99 (0.98, 1.01)	0.002 (0.017)
Sex at birth (female)	**1.37** ^***^ **(1.29, 1.45)**	**1.34** ^***^ **(1.27, 1.42)**	**1.47** ^***^ **(1.39, 1.55)**	0.95 (0.89, 1.01)	**2.09** ^***^ **(1.98, 2.21)**	**1.29** ^***^ **(1.21, 1.36)**	**1.212** ^***^ **(0.067)**
Residence (urban)	1.00 (0.94, 1.05)	1.03 (0.98, 1.09)	1.01 (0.96, 1.07)	**0.86** ^***^ **(0.81, 0.92)**	1.01 (0.95, 1.06)	1.01 (0.95, 1.07)	−0.321 (0.066)
Current annual family income	1.03 ^**^ (1.01, 1.05)	1.02 ^*^ (1.00, 1.04)	**1.10** ^***^ **(1.08, 1.12)**	**0.95** ^***^ **(0.92, 0.97)**	1.03 ^*^ (1.01, 1.05)	1.01 (0.99, 1.03)	−0.009 ^**^ (0.025)
Socioeconomic status	**0.90** ^***^ **(0.88, 0.91)**	**0.93** ^***^ **(0.91, 0.95)**	**0.93** ^***^ **(0.91, 0.94)**	0.99 (0.97, 1.00)	**0.97** ^***^ **(0.95, 0.98)**	**0.96** ^***^ **(0.94, 0.98)**	**−0.198** ^***^ **(0.020)**
Only child status (only child)	0.99 (0.94, 1.05)	1.04 (0.99, 1.10)	1.00 (0.94, 1.05)	1.05 (0.99, 1.12)	1.05 (0.99, 1.10)	1.03 (0.97, 1.09)	−0.264 (0.066)
Ethnicity (Han)	0.91 ^*^ (0.83, 0.99)	0.94 (0.87, 1.02)	0.95 (0.88, 1.03)	1.18 ^**^ (1.07, 1.30)	0.94 (0.87, 1.01)	0.95 (0.88, 1.04)	−0.129 ^*^ (0.098)
Smoking Status (yes)	**1.38** ^***^ **(1.29, 1.49)**	**1.29** ^***^ **(1.20, 1.38)**	1.04 (0.97, 1.12)	**0.74** ^***^ **(0.68, 0.81)**	**1.41** ^***^ **(1.31, 1.51)**	**1.60** ^***^ **(1.49, 1.71)**	**−0.845** ^***^ **(0.085)**
Alcohol consumption (yes)	**1.77** ^***^ **(1.55, 2.03)**	**1.58** ^***^ **(1.40, 1.78)**	**1.44** ^***^ **(1.27, 1.62)**	0.92 (0.79, 1.06)	**1.53** ^***^ **(1.36, 1.72)**	**1.66** ^***^ **(1.48, 1.87)**	**−0.284** ^***^ **(0.146)**
Exercise	**0.84** ^***^ **(0.82, 0.86)**	**0.87** ^***^ **(0.85, 0.89)**	**0.95** ^***^ **(0.92, 0.98)**	0.96 ^*^ (0.93, 0.99)	0.97 ^*^ (0.94, 1.00)	**0.89** ^***^ **(0.87, 0.92)**	**−0.545** ^***^ **(0.034)**
*CT family–related characteristics*							
Family type (ref: nuclear family)							
More than three generation	1.00 (0.93, 1.07)	1.04 (0.97, 1.11)	1.06 (0.99, 1.13)	0.99 (0.92, 1.07)	**1.13** ^***^ **(1.05, 1.20)**	1.13 ^**^ (1.05, 1.21)	0.071 (0.083)
Others	0.93 (0.86, 1.01)	0.91 ^**^ (0.84, 0.97)	0.93 ^*^ (0.87, 1.00)	0.95 (0.88, 1.03)	**1.14** ^***^ **(1.07, 1.22)**	1.04 (0.97, 1.12)	−0.165 (0.086)
Relationship with father	**1.15** ^***^ **(1.10, 1.20)**	**1.11** ^***^ **(1.07, 1.15)**	**1.15** ^***^ **(1.11, 1.19)**	1.04 ^*^ (1.00, 1.09)	1.03 (1.00, 1.07)	**1.11** ^***^ **(1.01, 1.15)**	**0.478** ^***^ **(0.043)**
Relationship with mother	**1.14** ^***^ **(1.09, 1.20)**	**1.09** ^***^ **(1.05, 1.14)**	**1.11** ^***^ **(1.07, 1.16)**	1.04 (0.99, 1.09)	0.95 ^*^ (0.92, 0.99)	1.04 ^*^ (1.00, 1.09)	**0.291** ^***^ **(0.049)**
Family harmony	**0.83** ^***^ **(0.82, 0.84)**	**0.85** ^***^ **(0.84, 0.87)**	**0.85** ^***^ **(0.84, 0.87)**	**1.03** ^***^ **(1.02, 1.05)**	**0.93** ^***^ **(0.91, 0.94)**	**0.88** ^***^ **(0.87, 0.89)**	**−0.356** ^***^ **(0.016)**
Education level of father	**1.04** ^***^ **(1.02, 1.06)**	**1.05** ^***^ **(1.03, 1.07)**	**1.05** ^***^ **(1.03, 1.06)**	1.01 (0.98, 1.03)	1.02 (1.00, 1.04)	**1.05** ^***^ **(1.03, 1.07)**	**0.086** ^***^ **(0.022)**
Education level of mother	0.99 (0.97, 1.01)	0.98 (0.97, 1.00)	1.00 (0.98, 1.02)	1.01 (0.99, 1.03)	0.98 ^**^ (0.96, 0.99)	0.98 (0.96, 1.00)	−0.065 (0.023)

*Note*: Hierarchical logistic regression was performed to find risk factors for six diseases: Depression, GAD, OCD, autism, eating disorder and PTSD according to the cutoff values of the corresponding scales. Since the scale of SAD has no definite cutoff value, hierarchical logistic regression cannot be performed, so hierarchical linear regression is adopted here. This table only presents the results of the second layer of regression. *P < 0.05, **P < 0.01, ***P < 0.001. Bold values refer to significant associations between the independent and dependent variables at α = 0.001.

*Abbreviations*: *b*, standardized regression coefficient; CI, confidence intervals; CT, childhood trauma; GAD, generalized anxiety disorder; OCD, obsessive–compulsive disorder; OR, odds ratios; PTSD, post-traumatic stress disorder; SAD, social anxiety disorder.

In the final hierarchical regressions, family-related factors in the CT groups (i.e., family type, relationship with father, relationship with mother, family harmony, education level of mother, education level of father) improved the goodness of fit of the sociodemographic model. Table 4 illustrates that the sociodemographic characteristics, including sex, socioeconomic status, smoking, alcohol consumption, exercise, as well as family factors, specifically, relationship with father, relationship with mother, family harmony and father’s education level, were significantly associated with the prevalence of current psychiatric symptoms among the CT population. Females were more likely to display current psychiatric symptoms, other than autism (i.e., depression, GAD, OCD, eating disorders, PTSD, SAD), particularly eating disorders (OR = 2.09, 95% CI = 1.98–2.21, *P* < 0.001). Participants with a higher socioeconomic status were also less prone to current psychiatric symptoms. Among the lifestyle variables, smoking status, consumption of alcohol and lack of exercise were established as risk factors for these current psychiatric symptoms in CT populations. In terms of family variables, both education level of the father and a poor relationship with parents were significantly associated with current psychiatric symptoms. Family harmony was a protective factor for six types of current psychiatric symptoms outside of autism (OR = 1.03, 95% CI = 1.02–1.05, *P* < 0.001).

## Discussion

This large cross-sectional study explored the lasting effects of CT by combining the self-reported psychiatric disorders and current psychiatric symptoms among Chinese young adults. Moreover, this study investigated the risk factors of current psychiatric symptoms among those with experiences of CT. The results showed that the prevalence of both self-reported psychiatric disorders and current psychiatric symptoms were significantly higher in youth with CT experiences. Furthermore, several sociodemographic factors (i.e., females, family disharmony and low socioeconomic status, lifestyle factors) were significantly associated with current psychiatric symptoms. These findings are crucial to better understand the effect of CT and ply appropriate interventions.

In this study, the prevalence of CT was 28.76% (95% CI: 28.47–29.04%) among Chinese young adults. This figure is lower than Brazil (34.3%; Bauer et al., 2022) and the meta-analysis from China (64.7%; Fu et al., 2018), but higher than in Germany (13.4%; Witt et al., 2017). In Brazil, the prospective birth cohort study reported that 1,154 (34.3%) of 3,367 children at age 11 years had been exposed to trauma (including CT and other traumas; Bauer et al., 2022). In China, based on a meta-analysis of nine articles, the pooled prevalence of CT was 64.7% (95% CI: 52.3%–75.6%) among Chinese college students (Fu et al., 2018). A large review of a series of meta-analyses, including 244 publications and 551 prevalence rates reported a prevalence of 127/1,000 for SA, 226/1000 for PA and 363/1,000 for EA (Stoltenborgh et al., 2015). This study focused on additional experiences of CT among young adults, including EA, EN, PA, PN and SA. Due to the lack of comprehensive studies, research exploring broader adverse childhood experiences (ACEs), childhood maltreatment and early life adversity (Boullier and Blair, 2018) can provide additional insight. These experiences would encompass any adverse or stressful experiences encountered during childhood, including maltreatment and other forms of adversity such as poverty, parental separation or divorce, chronic illness, natural disasters or exposure to community violence (Merrick et al., 2018; Liu et al., 2021; Madigan et al., 2023). A systematic review and meta-analysis of 206 studies reported the pooled prevalence of ACEs in young adults to be: 39.9% (95% CI: 29.8–49.2) experienced no ACE and 22.4% of youth did (95% CI: 14.1–30.6), everyone ACE for half a million adults (Madigan et al., 2023), while another meta-analysis found the lifetime prevalence of four or more ACEs was 53.9% (95% CI: 45.9–61.7) among unhoused individuals (Liu et al., 2021), suggesting a strong link to poverty. In 2011–2014, the prevalence of ACEs in a sample of 214,157 adult respondents, using eight categories of maltreatment, across 23 states, was 61.55% (Merrick et al., 2018). However, considering the various definitions of ACEs, CT and childhood maltreatment, the pooled prevalence of these experiences should be compared with uniform definitions and standards, especially in different countries.

Although the prevalence of CT found in this study was different from the several studies mentioned, this is due to different assessments of CT (CTQ VS. questions), ages of participants (young adults vs. children) and different cultural contexts across the studies. Additionally, individuals who experience CT may be reluctant to discuss their past experiences, leading to a lower reported prevalences of the condition (Pasupathi et al., 2009). Besides, in Jilin Province, which is primarily an agricultural region in China, some individuals may not be aware that they have experienced CT. In China’s cultural context, these youth might perceive occasional parental discipline as a normal occurrence. This lack of awareness may contribute to a certain degree of reduced report rates for CT.

The results showed that after controlling for confounding factors, the prevalence rates of self-reported psychiatric disorders and total scores of current psychiatric symptoms were significantly higher among the CT group. These results further demonstrated the sustained damage of CT upon mental health among young adults, which is consistent with previous researches. For example, several systematic reviews and meta-analyses indicate a significant association between childhood exposures (EA, PA and trauma exposure) and adult psychiatric disorders (McKay et al., 2021), between CT and lifetime suicide attempts risk (Zatti et al., 2017), between CT and anxiety, depression and substance disorders (McKay et al., 2022), as well as sleep disorders, PTSD, ADHD, SUDs and other psychiatric disorders (Chen et al., 2010; Mironova et al., 2011; Norman et al., 2012; Varese et al., 2012; Lindert et al., 2014; Hughes et al., 2017; Thabet, 2017). Notably, prospective cohort studies also have found that after adjusting for childhood risk factors, cumulative CT exposure is still related to higher rates of adult psychiatric outcomes, including anxiety, depression and SUDs (Copeland et al., 2018; Bauer et al., 2022). Our results support the associations between CT experiences and psychiatric disorders to some extent. The extant literature has suggested exposure to CT would cause neurological, physiological and psychological disruptions (Varese et al., 2012; Massullo et al., 2023). Experiences with CT could induce neurodevelopmental changes, such as dysregulation of the functioning of HPA, which plays a central role in the body’s response to stress (McKay et al., 2021). Several studies have found that adults with a history of childhood abuse demonstrate persistent sensitization of the pituitary–adrenal and autonomic stress response, which might increase the risk of psychiatric disorders among CT survivors (Lindert et al., 2014). Secondly, according to psychological theories, CT experiences might affect a child’s cognitive schema of themselves, others and the world, which leaves them vulnerable to negative beliefs (Dannlowski et al., 2012). These negative cognitions may affect the individual’s later susceptibility to psychiatric disorders such as depression, anxiety, PTSD and OCD, as well as sensitivity to rejection and abandonment, unstable relationships and difficulty with trust (Lindert et al., 2014). More seriously, CT has been found to affect the young person’s quality of life, and increase the risk of suicidal behaviors (Lindert et al., 2014; Zatti et al., 2017). Several studies have found that young adults who report a higher prevalence of suicidal ideation and suicidal attempts have experienced SA and PA (Hesdorffer et al., 2009). Considering the devastating effect of CT experiences on young adults, the mental health status of these individuals requires further attention and effective strategies are required to improve their quality of life.

According to these results, females with CT were more likely to report current psychiatric symptoms, which is consistent with previous studies (DeWit et al., 2005; Afifi et al., 2008; Tolin and Foa, 2008; Sweeney et al., 2015; Pruessner et al., 2019; Tsapenko, 2021; Bhattacharya and Sharan, 2022). Particularly, a near-term study suggested females with ACEs (e.g., SA, domestic violence) appeared to have more complex patterns of social and emotional difficulties, incurring mental health issues across the lifespan (Haahr-Pedersen et al., 2020). Several suggestions might explain these results. First, compared to males, females appear more vulnerable to acute emotional responses (Olff et al., 2007; e.g., intense fear, helplessness, horror, intrusive thoughts, avoidance, panic and anxiety) as well as acute dissociative responses, they are more likely to fall into rumination. These female youth would build a negative cognitive schema of repetitively and passively ruminating and reflecting on symptoms of distress, which increased the risk of psychiatric symptoms and disorders developing (Hesdorffer et al., 2009). Moreover, sex differences in coping styles exist when it comes to trauma, with males inclined to exhibit more problem-focused reactions to CT experiences, while females present with emotion-focused coping strategies (Sigurdardottir et al., 2014). Furthermore, sex hormones particularly progesterone (female dominant), might facilitate the development of anxiety and PTSD, especially as most youths experiencing puberty during the episode(s) of CT. All these factors could potentially explain why females were more likely to report current psychiatric symptoms when they have experienced CT.

In accordance with previous studies (Trinidad et al., 2003; Tylka and Kroon Van Diest, 2015; Thabet, 2017), young adults with CT experiences who were from harmonious family environments were less likely to suffer from current psychiatric symptoms. Substantial studies have found the important role of family harmony in a child’s development, particularly in the Chinese cultural context (Alink et al., 2009; Nursalam et al., 2009; Balistreri and Alvira-Hammond, 2016; Zhang et al., 2021). The perception of support and encouragement from family and the high levels of intimacy are particularly important for improving self-confidence and self-esteem and reducing negative emotions. For example, Zhang et al. (2021) explored the mediating roles of family functioning between CT and general distress, reporting that good family functioning could predict better emotional states. Additionally, studies also discovered that higher family social support brings higher self-esteem and more optimistic views, which is conducive to coping with problems positively. Furthermore, the study found that higher socioeconomic status is related to a lower risk of current psychiatric symptoms. The higher socioeconomic status usually is related to the higher educational levels of parents (Bradley and Corwyn, 2002), which is associated with higher empathy and providing familial support to their children when they experience CT. Higher socioeconomic status also comes with the resources to pay for professional help, given the high treatment fee in China and shortage of practitioners. All these factors could help young adults cope better with CT experiences and reduce the risk of psychiatric disorders and symptoms.

Apart from the above factors, lifestyle factors, including exercise, smoking status and alcohol consumption, are further associated with current psychiatric symptoms among those who have experienced CT. The advantage of exercise for improving mental health status has been well established, such as antidepressant effects and anxiolytic neurobiological effects (e.g., improved HPA axis functioning, increased monoamine neurotransmission), which are beneficial for adolescents to develop life skills (e.g., initiative, teamwork, self-control). Moreover, exercise provides a distraction from stressors, keeping individuals away from constant worry thereby reducing depression and anxiety (Salmon, 2001; Motta et al., 2012; Tessier et al., 2023). Smoking status and alcohol consumption have been suggested as harmful to the mental health of younger adults (Chang et al., 2005; Giannakopoulos et al., 2010; Barry et al., 2017; Tembo et al., 2017; Ferreira et al., 2019). Furthermore, youth with CT experiences would be more likely to smoke and drink as coping methods to relieve the negative effects of CT, with the associations between psychiatric symptoms, smoking or/and drinking strengthened in turn. Therefore, to improve the quality of life and mental health of youth with CT experiences, suitable lifestyle guidance should be recommended, such as more exercise, less smoking and less units of alcohol.

## Research and practical implications

Recognizing the lasting effects of CT on mental health, future interventions for young adults should adopt a comprehensive and tailored approach. First, programs should offer evidence-based therapeutic interventions, such as cognitive-behavioral therapy (CBT; Cohen and Mannarino, 2019), dialectical behavior therapy (DBT; Choi-Kain et al., 2021) and trauma-informed therapies, to address the complex interplay of trauma-based symptomology as well as additional mental health disorders. Moreover, interventions should prioritize early identification of CT and special attention should be given to at-risk children as a preventive measure to mental health issues later in life. This could involve implementing screening protocols in primary care settings, educational institutions and community organizations to identify children who may be experiencing CT. Furthermore, holistic support services should be established to address the diverse needs of young adults affected by CT later in life. This includes providing access to counseling services, psychiatric care, peer support groups and other resources aimed at promoting resilience and recovery in youths. Additionally, interventions should incorporate psychoeducation components to enhance young adults’ understanding of CT and its impact on mental health, empowering them to seek help and advocate for their needs. Collaboration between healthcare providers, educators, policymakers and community stakeholders is essential to ensure the successful implementation of these interventions. By working together, they can create a supportive and inclusive environment that promotes healing, resilience and holistic well-being for young adults affected by CT.

## Limitations of the study

Although this is a large-scale study to investigate the psychiatric impact of CT experiences among more than 110,000 college students in China, several limitations should be noted. First, CT experiences and previous psychiatric disorders were trusted as reported accurately by participants in terms of if they were actually diagnosed by psychiatrists, and the self-report by participants would have caused recall bias to some extent. In addition, the comorbid diagnoses of psychiatric disorders failed to be captured in this study. Longitudinal studies should be conducted to reduce recall bias and comorbidity needs to be considered in the design measures. Furthermore, due to the study design, several important moderators such as social support, self-esteem, self-reliance were not measured. The association between CT and psychiatric disorders required thorough exploration. Due to the cross-sectional design, causality between variables and psychiatric symptoms cannot be determined. Additionally, due to the general call for participants among college students, there may be an over- or underrepresentation of those with CT experiences, as individuals may be more or less likely to enroll in the study. Moreover, appropriate psychological counseling information is not enough to help participants mitigate mental health risks when collecting data from a large sample size. In the future, a supportive school environment is required to alleviate potential mental health challenges associated with traumatic experiences and prevent these from occurring in the first place. Finally, this large-scale study was conducted in Jilin province, thus, the generalization of results should be cautious and may not apply to other areas outside of China.

## Conclusion

In conclusion, this is the largest population-based, cross-sectional study that compared the status of self-reported psychiatric disorders and current psychiatric symptoms between young adults with and without CT experiences in China. The results indicated that individuals exposured to CT more likely to report serious long-term psychiatric symptoms among young adults. Several demographic factors should be considered risk factors for psychiatric symptoms among those with experiences of CT, which were being female, having a lower socioeconomic status, family disharmony, poor relationship with parents, lower father’s education level and unhealthy lifestyle factors (i.e., smoking status, consumption of alcohol and lack of exercise). The implications of these findings necessitate the development of targeted strategies by public health entities and academic institutions aimed at enhancing the mental health and overall quality of life for youth, with particular attention to those who have experienced CT.

## Supporting information

Jin et al. supplementary material Jin et al. supplementary material

## Data Availability

All requests should be sent to the corresponding author. Based on the scientific rigor of the proposal, the study authors will discuss all requests and decide whether data sharing is appropriate.

## Supplementary material

The supplementary material for this article can be found at http://doi.org/10.1017/gmh.2024.100.

## References

[r1] Adler NE, Epel ES, Castellazzo G and Ickovics JR (2000) Relationship of subjective and objective social status with psychological and physiological functioning: Preliminary data in healthy white women. Health Psychology 19(6), 586–592. 11129362 10.1037//0278-6133.19.6.586

[r2] Afifi TO, Enns MW, Cox BJ, Asmundson GJ, Stein MB and Sareen J (2008) Population attributable fractions of psychiatric disorders and suicide ideation and attempts associated with adverse childhood experiences. American Journal of Public Health 98(5), 946–952. 18381992 10.2105/AJPH.2007.120253 PMC2374808

[r3] Alexander N, Kirschbaum C, Wankerl M, Stauch BJ, Stalder T, Steudte-Schmiedgen S, Muehlhan M and Miller R (2018) Glucocorticoid receptor gene methylation moderates the association of childhood trauma and cortisol stress reactivity. Psychoneuroendocrinology 90, 68–75. 29433075 10.1016/j.psyneuen.2018.01.020

[r4] Alink LR, Cicchetti D, Kim J and Rogosch FA (2009) Mediating and moderating processes in the relation between maltreatment and psychopathology: Mother-child relationship quality and emotion regulation. Journal of Abnormal Child Psychology 37(6), 831–843. 19301118 10.1007/s10802-009-9314-4 PMC2708329

[r5] Allison C, Auyeung B and Baron-Cohen S (2012) Toward brief “red flags” for autism screening: The short autism spectrum quotient and the short quantitative checklist in 1,000 cases and 3,000 controls. Journal of the American Academy of Child & Adolescent Psychiatry 51(2), 202–212.e7. 22265366 10.1016/j.jaac.2011.11.003

[r6] Assogna F, Piras F and Spalletta G (2020) Neurobiological basis of childhood trauma and the risk for neurological deficits later in life. In Childhood Trauma in Mental Disorders: A Comprehensive Approach. Cham: Springer, pp. 385–410.

[r7] Austin PC (2011) Optimal caliper widths for propensity‐score matching when estimating differences in means and differences in proportions in observational studies. Pharmaceutical Statistics 10(2), 150–161. 20925139 10.1002/pst.433 PMC3120982

[r8] Balistreri KS and Alvira-Hammond M (2016) Adverse childhood experiences, family functioning and adolescent health and emotional well-being. Public Health 132, 72–78. 26718424 10.1016/j.puhe.2015.10.034 PMC4798868

[r9] Barry AE, Jackson Z, Watkins DC, Goodwill JR and Hunte HE (2017) Alcohol use and mental health conditions among black college males: Do those attending postsecondary minority institutions fare better than those at primarily white institutions? American Journal of Men’s Health 11(4), 962–968. 10.1177/1557988316674840 PMC5675337 27807223

[r10] Bauer A, Fairchild G, Hammerton G, Murray J, Santos IS, Tovo Rodrigues L, Munhoz TN, Barros AJD, Matijasevich A and Halligan SL (2022) Associations between childhood trauma and childhood psychiatric disorders in Brazil: A population-based, prospective birth cohort study. Lancet Psychiatry 9(12), 969–977. 36328032 10.1016/S2215-0366(22)00337-6

[r11] Bernstein DP, Stein JA, Newcomb MD, Walker E, Pogge D, Ahluvalia T, Stokes J, Handelsman L, Medrano M and Desmond D (2003) Development and validation of a brief screening version of the childhood trauma questionnaire. Child Abuse & Neglect 27(2), 169–190. 12615092 10.1016/s0145-2134(02)00541-0

[r12] Bhattacharya M and Sharan AM (2022) Relationship of childhood sexual abuse with obsessive compulsive disorder during adulthood. Journal of Positive School Psychology 6(2), 4479–4491.

[r81] Boullier M, Blair ME (2018) Adverse childhood experiences. Paediatrics and Child Health, 28: 132–137.

[r13] Bradley RH and Corwyn RF (2002) Socioeconomic status and child development. Annual Review of Psychology 53(1), 371–399. 10.1146/annurev.psych.53.100901.135233 11752490

[r14] Chang G, Sherritt L and Knight JR (2005) Adolescent cigarette smoking and mental health symptoms. Journal of Adolescent Health 36(6), 517–522. 10.1016/j.jadohealth.2004.05.008 15901517

[r15] Chen LP, Murad MH, Paras ML, Colbenson KM, Sattler AL, Goranson EN, Elamin MB, Seime RJ, Shinozaki G, Prokop LJ and Zirakzadeh A (2010) Sexual abuse and lifetime diagnosis of psychiatric disorders: Systematic review and meta-analysis. Mayo Clinic Proceedings 85(7), 618–629. 20458101 10.4065/mcp.2009.0583 PMC2894717

[r82] Choi-Kain L, Wilks CR, Ilagan GS, Iliakis BA (2021) Dialectical behavior therapy for early life trauma. Current Treatment Options in Psychiatry, 8, 111–124. 10.1007/s40501-021-00242-2.

[r83] Cohen JA, Mannarino AP (2019) Trauma-focused cognitive behavioral therapy for childhood traumatic separation. Child Abuse & Neglect, 92, 179–195. 10.1016/j.chiabu.2019.03.006. 30999167

[r16] Copeland WE, Shanahan L, Hinesley J, Chan RF, Aberg KA, Fairbank JA, van den Oord EJCG and Costello EJ (2018) Association of childhood trauma exposure with adult psychiatric disorders and functional outcomes. JAMA Network Open 1(7), e184493–e184493. 30646356 10.1001/jamanetworkopen.2018.4493 PMC6324370

[r17] Dannlowski U, Stuhrmann A, Beutelmann V, Zwanzger P, Lenzen T, Grotegerd D, Domschke K, Hohoff C, Ohrmann P, Bauer J, Lindner C, Postert C, Konrad C, Arolt V, Heindel W, Suslow T and Kugel H (2012) Limbic scars: Long-term consequences of childhood maltreatment revealed by functional and structural magnetic resonance imaging. Biological Psychiatry 71(4), 286–293. 22112927 10.1016/j.biopsych.2011.10.021

[r18] DeWit DJ, Chandler-Coutts M, Offord DR, King G, McDougall J, Specht J and Stewart S (2005) Gender differences in the effects of family adversity on the risk of onset of DSM-III-R social phobia. Journal of Anxiety Disorders 19(5), 479–502. 15749569 10.1016/j.janxdis.2004.04.010

[r19] Eilertsen T, Hansen B, Kvale G, Abramowitz JS, Holm SE and Solem S (2017) The dimensional obsessive-compulsive scale: Development and validation of a short form (DOCS-SF). Frontiers in Psychology 8, 1503. 28928693 10.3389/fpsyg.2017.01503 PMC5591872

[r84] Fang X, Fry DA, Ji K, Finkelhor D, Chen J, Lannen P, Dunne MP (2015). The burden of child maltreatment in China: a systematic review. Bulletin of the World Health Organization 93(3), 176–185. 10.2471/BLT.14.140970. 25838613 PMC4371492

[r20] Fenigstein A, Scheier MF and Buss AH (1975) Public and private self-consciousness: Assessment and theory. Journal of Consulting and Clinical Psychology 43(4), 522.

[r21] Ferreira VR, Jardim TV, Sousa ALL, Rosa BMC and Jardim PCV (2019) Smoking, alcohol consumption and mental health: Data from the Brazilian study of cardiovascular risks in adolescents (ERICA). Addictive Behaviors Reports 9, 100147. 31193769 10.1016/j.abrep.2018.100147 PMC6542299

[r22] Foa EB, Riggs DS, Dancu CV and Rothbaum BO (1993) Reliability and validity of a brief instrument for assessing post‐traumatic stress disorder. Journal of Traumatic Stress 6(4), 459–473.

[r85] Fry D, McCoy A, Swales D (2012) The consequences of maltreatment on children’s lives: a systematic review of data from the East Asia and Pacific Region. Trauma Violence Abuse 13(4), 209–233. 10.1177/1524838012455873. 22899705

[r23] Fu H, Feng T, Qin J, Wang T, Wu X, Cai Y, Lan L and Yang T (2018) Reported prevalence of childhood maltreatment among Chinese college students: A systematic review and meta-analysis. PLoS One 13(10), e0205808. 30321243 10.1371/journal.pone.0205808 PMC6188789

[r24] Giannakopoulos G, Tzavara C, Dimitrakaki C, Kolaitis G, Rotsika V and Tountas Y (2010) Emotional, behavioural problems and cigarette smoking in adolescence: Findings of a Greek cross-sectional study. BMC Public Health 10, 1–7. 20128920 10.1186/1471-2458-10-57 PMC2835687

[r25] Haahr-Pedersen I, Perera C, Hyland P, Vallières F, Murphy D, Hansen M, Spitz P, Hansen P and Cloitre M (2020) Females have more complex patterns of childhood adversity: Implications for mental, social, and emotional outcomes in adulthood. European Journal of Psychotraumatology 11(1), 1708618. 32002142 10.1080/20008198.2019.1708618 PMC6968572

[r26] He J, Zhong X, Gao Y, Xiong G and Yao S (2019) Psychometric properties of the Chinese version of the childhood trauma questionnaire-short form (CTQ-SF) among undergraduates and depressive patients. Child Abuse & Neglect 91, 102–108. 30856597 10.1016/j.chiabu.2019.03.009

[r27] He X, Li C, Qian J, Cui H and Wu W (2010) Reliability and validity of a generalized anxiety disorder scale in general hospital outpatients. Shanghai Archives of Psychiatry 22(4), 200–203.

[r28] Hesdorffer DC, Rauch SL and Tamminga CA (2009) Long-term psychiatric outcomes following traumatic brain injury: A review of the literature. Journal of Head Trauma Rehabilitation 24(6), 452–459. 19940678 10.1097/HTR.0b013e3181c133fd

[r29] Horan JM and Widom CS (2015) From childhood maltreatment to allostatic load in adulthood: The role of social support. Child Maltreament 20(4), 229–239. 10.1177/1077559515597063 PMC5278954 26260146

[r30] Hughes K, Bellis MA, Hardcastle KA, Sethi D, Butchart A, Mikton C, Jones L and Dunne MP (2017) The effect of multiple adverse childhood experiences on health: A systematic review and meta-analysis. The Lancet Public Health 2(8), e356–e366. 29253477 10.1016/S2468-2667(17)30118-4

[r31] Jaffee SR and Maikovich-Fong AK (2011) Effects of chronic maltreatment and maltreatment timing on children’s behavior and cognitive abilities. The Journal of Child Psychology and Psychiatry 52(2), 184–194. 20735512 10.1111/j.1469-7610.2010.02304.x PMC2998571

[r32] Kisely S, Strathearn L and Najman JM (2020) Child maltreatment and mental health problems in 30-year-old adults: A birth cohort study. Journal of Psychiatric Research 129, 111–117. 32653613 10.1016/j.jpsychires.2020.06.009

[r33] Knipscheer J, Sleijpen M, Frank L, de Graaf R, Kleber R, Ten Have M and Dückers M (2020) Prevalence of potentially traumatic events, other life events and subsequent reactions indicative for posttraumatic stress disorder in the Netherlands: A general population study based on the Trauma Screening Questionnaire. International Journal of Environmental Research and Public Health 17(5), 1725. 32155752 10.3390/ijerph17051725 PMC7084195

[r34] Kroenke K, Spitzer RL and Williams JBW (2001) The PHQ-9. Journal of General Internal Medicine 16(9), 606–613. 11556941 10.1046/j.1525-1497.2001.016009606.x PMC1495268

[r35] Kutz AM, Marsh AG, Gunderson CG, Maguen S and Masheb RM (2020) Eating disorder screening: A systematic review and meta-analysis of diagnostic test characteristics of the SCOFF. Journal of General Internal Medicine 35, 885–893. 31705473 10.1007/s11606-019-05478-6 PMC7080881

[r36] Leung CN, Leung CSY, Chan RW and Leung PW (2023) Can the UK AQ‐10 be applicable to Chinese samples with autism spectrum disorder in Hong Kong? Cross‐cultural similarities and differences. Autism Research 16(2), 302–314. 36333966 10.1002/aur.2847

[r37] Lindert J, von Ehrenstein OS, Grashow R, Gal G, Braehler E and Weisskopf MG (2014) Sexual and physical abuse in childhood is associated with depression and anxiety over the life course: Systematic review and meta-analysis. International Journal of Public Health 59(2), 359–372. 24122075 10.1007/s00038-013-0519-5

[r86] Liu J, Wu J, Liu S, Li M, Hu K, Li K (2021). Predicting mortality of patients with acute kidney injury in the ICU using XGBoost Model. PLoS One, 16(2), e0246306. 33539390 10.1371/journal.pone.0246306 PMC7861386

[r87] Madigan S, Racine N, Vaillancourt T, Korczak DJ, Hewitt JMA, Pador P, Park JL, McArthur BA, Holy C, Neville RD (2023) Changes in depression and anxiety among children and adolescents from before to during the COVID-19 pandemic: a systematic review and meta-analysis. JAMA Pediatrics, 177(6), 567–581. 10.1001/jamapediatrics.2023.0846. 37126337 PMC10152379

[r38] Massullo C, De Rossi E, Carbone GA, Imperatori C, Ardito RB, Adenzato M and Farina B (2023) Child maltreatment, abuse, and neglect: An umbrella review of their prevalence and definitions. Clinical Neuropsychiatry 20(2), 72–99. 37250758 10.36131/cnfioritieditore20230201 PMC10211430

[r39] McKay MT, Cannon M, Chambers D, Conroy RM, Coughlan H, Dodd P, Healy C, O’Donnell L and Clarke MC (2021) Childhood trauma and adult mental disorder: A systematic review and meta-analysis of longitudinal cohort studies. Acta Psychiatrica Scandinavica 143(3), 189–205. 33315268 10.1111/acps.13268

[r40] McKay MT, Kilmartin L, Meagher A, Cannon M, Healy C and Clarke MC (2022) A revised and extended systematic review and meta-analysis of the relationship between childhood adversity and adult psychiatric disorder. Journal of Psychiatric Research 156, 268–283. 36274532 10.1016/j.jpsychires.2022.10.015

[r88] Merrick MT, Ford DC, Ports KA, Guinn AS (2018). Prevalence of adverse childhood experiences from the 2011–2014 behavioral risk factor surveillance system in 23 states. JAMA Pediatrics, 172(11), 1038–1044. 10.1001/jamapediatrics.2018.2537. 30242348 PMC6248156

[r41] Mironova P, Rhodes AE, Bethell JM, Tonmyr L, Boyle MH, Wekerle C, Goodman D and Leslie B (2011) Childhood physical abuse and suicide-related behavior: A systematic review. Vulnerable Children and Youth Studies 6(1), 1–7.

[r42] Morgan JF, Reid F and Lacey JH (1999) The SCOFF questionnaire: Assessment of a new screening tool for eating disorders. BMJ 319(7223), 1467–1468. 10582927 10.1136/bmj.319.7223.1467 PMC28290

[r43] Motta RW, McWilliams ME, Schwartz JT and Cavera RS (2012) The role of exercise in reducing childhood and adolescent PTSD, anxiety, and depression. Journal of Applied School Psychology 28(3), 224–238.

[r44] Nemeroff CB (2004) Neurobiological consequences of childhood trauma. Journal of Clinical Psychiatry 65, 18–28. 14728093

[r45] Norman RE, Byambaa M, De R, Butchart A, Scott J and Vos T (2012) The long-term health consequences of child physical abuse, emotional abuse, and neglect: A systematic review and meta-analysis. PLoS Medicine 9(11), e1001349. 23209385 10.1371/journal.pmed.1001349 PMC3507962

[r46] Nursalam N, Ni Ketut Alit A and Rista F (2009) Family social support reduces post judegemental stress in teenagers. Jurnal Ners 4(2), 182–189.

[r47] Olff M, Langeland W, Draijer N and Gersons BP (2007) Gender differences in posttraumatic stress disorder. Psychological Bulletin 133(2), 183. 17338596 10.1037/0033-2909.133.2.183

[r48] Park C, Park IH, Yoo T, Kim H, Ryu S, Lee JY, Kim JM and Kim SW (2021) Association between childhood trauma and suicidal behavior in the general population. Chonnam Medicine Journal 57(2), 126–131. 10.4068/cmj.2021.57.2.126 PMC8167439 34123740

[r49] Pasupathi M, McLean KC and Weeks T (2009) To tell or not to tell: Disclosure and the narrative self. Journal of Personality 77(1), 89–124. 19076992 10.1111/j.1467-6494.2008.00539.x

[r50] Petruccelli K, Davis J and Berman T (2019) Adverse childhood experiences and associated health outcomes: A systematic review and meta-analysis. Child Abuse & Neglect 97, 104127. 31454589 10.1016/j.chiabu.2019.104127

[r51] Pruessner M, King S, Vracotas N, Abadi S, Iyer S, Malla AK, Shah J and Joober R (2019) Gender differences in childhood trauma in first episode psychosis: Association with symptom severity over two years. Schizophrenia Research 205, 30–37. 29935881 10.1016/j.schres.2018.06.043

[r52] Read J, van Os J, Morrison AP and Ross CA (2005) Childhood trauma, psychosis and schizophrenia: A literature review with theoretical and clinical implications. Acta Psychiatrica Scandinavica 112(5), 330–350. 16223421 10.1111/j.1600-0447.2005.00634.x

[r53] Salmon P (2001) Effects of physical exercise on anxiety, depression, and sensitivity to stress: A unifying theory. Clinical Psychology Review 21(1), 33–61. 11148895 10.1016/s0272-7358(99)00032-x

[r54] Shek DT (1994) Assessment of private and public self‐consciousness: A Chinese replication. Journal of Clinical Psychology 50(3), 341–348. 8071439 10.1002/1097-4679(199405)50:3<341::aid-jclp2270500305>3.0.co;2-t

[r55] Sigurdardottir S, Halldorsdottir S and Bender SS (2014) Consequences of childhood sexual abuse for health and well-being: Gender similarities and differences. Scandinavian Journal of Public Health 42(3), 278–286. 24345814 10.1177/1403494813514645

[r56] Spitzer RL, Kroenke K, Williams JBW and Löwe B (2006) A brief measure for assessing generalized anxiety disorder: The GAD-7. Archives of Internal Medicine 166(10), 1092–1097. 16717171 10.1001/archinte.166.10.1092

[r89] Stoltenborgh M, Bakermans-Kranenburg MJ, Alink LRA, van IJzendoorn MH (2015). The prevalence of child maltreatment across the globe: review of a series of meta-analyses.

[r57] Sweeney S, Air T, Zannettino L, Shah SS and Galletly C (2015) Gender differences in the physical and psychological manifestation of childhood trauma and/or adversity in people with psychosis. Frontiers in Psychology 6, 1768. 26635676 10.3389/fpsyg.2015.01768 PMC4655246

[r58] Tembo C, Burns S and Kalembo F (2017) The association between levels of alcohol consumption and mental health problems and academic performance among young university students. PLoS One 12(6), e0178142. 28658300 10.1371/journal.pone.0178142 PMC5489147

[r59] Tessier AJ, Moyen A, Lawson C, Rappaport AI, Yousif H, Fleurent-Grégoire C, Lalonde-Bester S, Brazeau AS and Chevalier S (2023) Lifestyle behavior changes and associated risk factors during the COVID-19 pandemic: Results from the Canadian COVIDiet online cohort study. JMIR Public Health and Surveillence 9, e43786. 10.2196/43786 PMC10131911 36848226

[r60] Thabet AAM (2017) Risk and protective factors in relation to trauma and post traumatic stress disorders: A meta-analytic review. EC Psychology and Psychiatry 2(4), 122–138.

[r61] Tolin DF and Foa EB (2008) Sex differences in trauma and posttraumatic stress disorder: A quantitative review of 25 years of research. Psychological Bulletin 132(6), 959–992. 10.1037/0033-2909.132.6.959 17073529

[r62] Trinidad DR, Chou C-P, Unger JB, Anderson Johnson C and Li Y (2003) Family harmony as a protective factor against adolescent tobacco and alcohol use in Wuhan, China. Substance Use & Misuse 38(8), 1159–1171. 12901453 10.1081/ja-120017656

[r63] Tsapenko A (2021) Traumatic influence of the attitude towards one’s own gender as a factor in the development of eating disorders. E3S Web of Conferences 273, 10046.

[r64] Tylka TL and Kroon Van Diest AM (2015) Protective factors. In The Wiley Handbook of Eating Disorders. Sussex, UK: John Wiley & Sons, pp. 430–444.

[r65] Varese F, Smeets F, Drukker M, Lieverse R, Lataster T, Viechtbauer W, Read J, van Os J and Bentall RP (2012) Childhood adversities increase the risk of psychosis: A meta-analysis of patient-control, prospective- and cross-sectional cohort studies. Schizophrenia Bulletin 38(4), 661–671. 22461484 10.1093/schbul/sbs050 PMC3406538

[r66] Von Elm E, Altman DG, Egger M, Pocock SJ, Gøtzsche PC and Vandenbroucke JP (2007) The strengthening the reporting of observational studies in epidemiology (STROBE) statement: Guidelines for reporting observational studies. The Lancet 370(9596), 1453–1457. 10.1016/S0140-6736(07)61602-X 18064739

[r67] Walters JT, Bisson JI and Shepherd JP (2007) Predicting post-traumatic stress disorder: Validation of the Trauma Screening Questionnaire in victims of assault. Psychological Medicine 37(1), 143–150. 16959058 10.1017/S0033291706008658

[r68] Wang W, Bian Q, Zhao Y, Li X, Wang W, Du J, Zhang G, Zhou Q and Zhao M (2014) Reliability and validity of the Chinese version of the patient health questionnaire (PHQ-9) in the general population. General Hospital Psychiatry 36(5), 539–544. 25023953 10.1016/j.genhosppsych.2014.05.021

[r69] Witt A, Brown RC, Plener PL, Brähler E and Fegert JM (2017) Child maltreatment in Germany: Prevalence rates in the general population. Child and Adolescent Psychiatry and Mental Health 11, 47. 28974983 10.1186/s13034-017-0185-0 PMC5621113

[r70] World Health Organization (2014) *Violence against Children.* Available at https://apps.who.int/violence-info/child-maltreatment (accessed 17 October).

[r71] World Medical Association (2013) World medical association declaration of Helsinki: Ethical principles for medical research involving human subjects. JAMA 310(20), 2191–2194. 24141714 10.1001/jama.2013.281053

[r72] Wu KK (2014) PTSD and loss: Preliminary findings from a territory-wide epidemiology study in Hong Kong. European Journal of Psychotraumatology 5(1), 26517. 25511722 10.3402/ejpt.v5.26517 PMC4265181

[r73] Wu KK, Leung PW, Wong CS, Yu PM, Luk BT, Cheng JP, Wong RM, Wong PP, Lui JC and Ngan JC (2019) The Hong Kong survey on the epidemiology of trauma exposure and posttraumatic stress disorder. Journal of Traumatic Stress 32(5), 664–676. 31393657 10.1002/jts.22430

[r74] Wu NS, Schairer LC, Dellor E and Grella C (2010) Childhood trauma and health outcomes in adults with comorbid substance abuse and mental health disorders. Addictive Behaviors 35(1), 68–71. 19775820 10.1016/j.addbeh.2009.09.003 PMC3666315

[r75] Xiao Z, Murat Baldwin M, Wong SC, Obsuth I, Meinck F and Murray AL (2023) The impact of childhood psychological maltreatment on mental health outcomes in adulthood: A systematic review and meta-analysis. Trauma Violence Abuse 24(5), 3049–3064. 36123796 10.1177/15248380221122816 PMC10594835

[r76] Xiaona X and Xiaoping L (2018) The effect of subjective social class on prosocial behavior. Studies of Psychology and Behavior 16(4), 563.

[r77] Zatti C, Rosa V, Barros A, Valdivia L, Calegaro VC, Freitas LH, Ceresér KMM, Rocha NSD, Bastos AG and Schuch FB (2017) Childhood trauma and suicide attempt: A meta-analysis of longitudinal studies from the last decade. Psychiatry Research 256, 353–358. 28683433 10.1016/j.psychres.2017.06.082

[r78] Zhang L, Ma X, Yu X, Ye M, Li N, Lu S and Wang J (2021) Childhood trauma and psychological distress: A serial mediation model among Chinese adolescents. International Journal of Environmental Research and Public Health 18(13), 6808. 34202902 10.3390/ijerph18136808 PMC8297141

[r79] Zhang Z, Kim HJ, Lonjon G and Zhu Y (2019) Balance diagnostics after propensity score matching. Annals of Translational Medicine 7(1), 16. 30788363 10.21037/atm.2018.12.10 PMC6351359

