# Supplementary material for: The lasting effects of childhood trauma on developing psychiatric symptoms: A population-based, large-scale comparison study
Source: Glob Ment Health (Camb). 2024 Oct 24;11:e98. doi: 10.1017/gmh.2024.100 (PMC11504938; doi:10.1017/gmh.2024.100)
Supplement: Jin et al. supplementary material [file S2054425124001006sup001.docx]

**Supplementary material**

Table S1. Overall outcomes from hierarchical regression analysis.

Table S1. Overall outcomes from hierarchical regression analysis.

| Independent Variable | Depression | | | |
| --- | --- | --- | --- | --- |
|  | Model1 | | Model2 | |
|  | OR value (95% CI) | *P* value | OR value (95% CI) | *P* value |
| Age | 1.00 (0.98,1.01) | 0.533 | 0.99 (0.98,1.01) | 0.337 |
| Sex at birth |  |  |  |  |
| Female (Ref = Male) | **1.56 (1.48,1.65)** | **<0.001** | **1.37 (1.29,1.45)** | **<0.001** |
| Residence |  |  |  |  |
| Urban (Ref = Rural) | 1.05 (1.00,1.11) | 0.074 | 1.00 (0.94,1.05) | 0.890 |
| Current annual family income | **1.04 (1.02,1.07)** | **<0.001** | 1.03 (1.01,1.05) | 0.006 |
| Socioeconomic status | **0.84 (0.83,0.86)** | **<0.001** | **0.90 (0.88,0.91)** | **<0.001** |
| Only child status |  |  |  |  |
| Yes (Ref = No) | 0.97 (0.92,1.02) | 0.194 | 0.99 (0.94,1.05) | 0.730 |
| Ethnicity |  |  |  |  |
| Han (Ref = non-Han Chinese) | 0.90 (0.83,0.98) | 0.013 | 0.91 (0.83,0.99) | 0.024 |
| Smoking |  |  |  |  |
| Yes (Ref = No) | **1.32 (1.23,1.42)** | **<0.001** | **1.38 (1.29,1.49)** | **<0.001** |
| Alcohol Consumption |  |  |  |  |
| Yes (Ref = No) | **1.72 (1.51,1.97)** | **<0.001** | **1.77 (1.55,2.03)** | **<0.001** |
| Exercise | **0.81 (0.79,0.83)** | **<0.001** | **0.84 (0.82,0.86)** | **<0.001** |
| Family type (Ref = Nuclear family) |  |  |  |  |
| More than three generation |  |  | 1.00 (0.93,1.07) | 0.892 |
| Others |  |  | 0.93 (0.86,1.01) | 0.066 |
| Relationship with father |  |  | **1.15 (1.10,1.20)** | **<0.001** |
| Relationship with mother |  |  | **1.14 (1.09,1.20)** | **<0.001** |
| Family harmony |  |  | **0.83 (0.82,0.84)** | **<0.001** |
| Education level of mother |  |  | **1.04 (1.02,1.06)** | **<0.001** |
| Education level of father |  |  | 0.99 (0.97,1.01) | 0.219 |

| Independent Variable | GAD | | | |
| --- | --- | --- | --- | --- |
|  | Model1 | | Model2 | |
|  | OR value (95% CI) | *P* value | OR value (95% CI) | *P* value |
| Age | 0.99 (0.98,1.01) | 0.216 | 0.99 (0.98,1.00) | 0.112 |
| Sex at birth |  |  |  |  |
| Female (Ref = Male) | **1.51 (1.43,1.59)** | **<0.001** | **1.34 (1.27,1.42)** | **<0.001** |
| Residence |  |  |  |  |
| Urban (Ref = Rural) | 1.08 (1.02,1.13) | 0.005 | 1.03 (0.98,1.09) | 0.281 |
| Current annual family income | 1.04 (1.02,1.06) | 0.001 | 1.02 (1.00,1.04) | 0.035 |
| Socioeconomic status | **0.88 (0.87,0.89)** | **<0.001** | **0.93 (0.91,0.95)** | **<0.001** |
| Only child status |  |  |  |  |
| Yes (Ref = No) | 1.02 (0.97,1.07) | 0.487 | 1.04 (0.99,1.10) | 0.157 |
| Ethnicity |  |  |  |  |
| Han (Ref = non-Han Chinese) | 0.93 (0.86,1.01) | 0.068 | 0.94 (0.87,1.02) | 0.110 |
| Smoking |  |  |  |  |
| Yes (Ref = No) | **1.25 (1.17,1.34)** | **<0.001** | **1.29 (1.20,1.38)** | **<0.001** |
| Alcohol Consumption |  |  |  |  |
| Yes (Ref = No) | **1.56 (1.39,1.75)** | **<0.001** | **1.58 (1.40,1.78)** | **<0.001** |
| Exercise | **0.84 (0.82,0.86)** | **<0.001** | **0.87 (0.85,0.89)** | **<0.001** |
| Family type (Ref = Nuclear family) |  |  |  |  |
| More than three generation |  |  | 1.04 (0.97,1.11) | 0.231 |
| Others |  |  | 0.91 (0.84,0.97) | 0.005 |
| Relationship with father |  |  | **1.11 (1.07,1.15)** | **<0.001** |
| Relationship with mother |  |  | **1.09 (1.05,1.14)** | **<0.001** |
| Family harmony |  |  | **0.85 (0.84,0.87)** | **<0.001** |
| Education level of mother |  |  | **1.05 (1.03,1.07)** | **<0.001** |
| Education level of father |  |  | 0.98 (0.97,1.00) | 0.071 |

| Independent Variable | OCD | | | |
| --- | --- | --- | --- | --- |
|  | Model1 | | Model2 | |
|  | OR value (95% CI) | *P* value | OR value (95% CI) | *P* value |
| Age | 1.00 (0.98,1.01) | 0.754 | 1.00 (0.98,1.01) | 0.504 |
| Sex at birth |  |  |  |  |
| Female (Ref = Male) | **1.65 (1.57,1.74)** | **<0.001** | **1.47 (1.39,1.55)** | **<0.001** |
| Residence |  |  |  |  |
| Urban (Ref = Rural) | 1.07 (1.01,1.12) | 0.013 | 1.01 (0.96,1.07) | 0.681 |
| Current annual family income | **1.11 (1.09,1.14)** | **<0.001** | **1.10 (1.08,1.12)** | **<0.001** |
| Socioeconomic status | **0.87 (0.86,0.89)** | **<0.001** | **0.93 (0.91,0.94)** | **<0.001** |
| Only child status |  |  |  |  |
| Yes (Ref = No) | 0.99 (0.94,1.04) | 0.618 | 1.00 (0.94,1.05) | 0.899 |
| Ethnicity |  |  |  |  |
| Han (Ref = non-Han Chinese) | 0.94 (0.87,1.02) | 0.129 | 0.95 (0.88,1.03) | 0.206 |
| Smoking |  |  |  |  |
| Yes (Ref = No) | 1.03 (0.96,1.10) | 0.482 | 1.04 (0.97,1.12) | 0.297 |
| Alcohol Consumption |  |  |  |  |
| Yes (Ref = No) | **1.43 (1.27,1.60)** | **<0.001** | **1.44 (1.27,1.62)** | **<0.001** |
| Exercise | **0.91 (0.89,0.94)** | **<0.001** | **0.95 (0.92,0.98)** | **<0.001** |
| Family type (Ref = Nuclear family) |  |  |  |  |
| More than three generation |  |  | 1.06(0.99,1.13) | 0.115 |
| Others |  |  | 0.93 (0.87,1.00) | 0.044 |
| Relationship with father |  |  | **1.15 (1.11,1.19)** | **<0.001** |
| Relationship with mother |  |  | **1.11 (1.07,1.16)** | **<0.001** |
| Family harmony |  |  | **0.85 (0.84,0.87)** | **<0.001** |
| Education level of mother |  |  | **1.05 (1.03,1.06)** | **<0.001** |
| Education level of father |  |  | 1.00 (0.98,1.02) | 0.961 |

| Independent Variable | Autism | | | |
| --- | --- | --- | --- | --- |
|  | Model1 | | Model2 | |
|  | OR value (95% CI) | *P* value | OR value (95% CI) | *P* value |
| Age | 1.01 (1.00,1.03) | 0.083 | 1.02 (1.00,1.03) | 0.076 |
| Sex at birth |  |  |  |  |
| Female (Ref = Male) | 0.94 (0.88,1.00) | 0.036 | 0.95 (0.89,1.01) | 0.096 |
| Residence |  |  |  |  |
| Urban (Ref = Rural) | **0.87 (0.82,0.92)** | **<0.001** | **0.86 (0.81,0.92)** | **<0.001** |
| Current annual family income | **0.95 (0.93,0.97)** | **<0.001** | **0.95 (0.92,0.97)** | **<0.001** |
| Socioeconomic status | 0.99 (0.97,1.01) | 0.334 | 0.99 (0.97,1.00) | 0.112 |
| Only child status |  |  |  |  |
| Yes (Ref = No) | 1.05 (0.99,1.11) | 0.125 | 1.05 (0.99,1.12) | 0.123 |
| Ethnicity |  |  |  |  |
| Han (Ref = non-Han Chinese) | **1.19 (1.08,1.31)** | **<0.001** | 1.18 (1.07,1.30) | 0.001 |
| Smoking |  |  |  |  |
| Yes (Ref = No) | **0.74 (0.68,0.80)** | **<0.001** | **0.74 (0.68,0.81)** | **<0.001** |
| Alcohol Consumption |  |  |  |  |
| Yes (Ref = No) | 0.92 (0.79,1.06) | 0.255 | 0.92 (0.79,1.06) | 0.260 |
| Exercise | 0.96 (0.93,0.99) | 0.016 | 0.96 (0.93,0.99) | 0.011 |
| Family type (Ref = Nuclear family) |  |  |  |  |
| More than three generation |  |  | 0.99 (0.92,1.07) | 0.800 |
| Others |  |  | 0.95 (0.88,1.03) | 0.233 |
| Relationship with father |  |  | 1.04 (1.00,1.09) | 0.042 |
| Relationship with mother |  |  | 1.04 (0.99,1.09) | 0.118 |
| Family harmony |  |  | **1.03 (1.02,1.05)** | **<0.001** |
| Education level of mother |  |  | 1.01 (0.98,1.03) | 0.668 |
| Education level of father |  |  | 1.01 (0.99,1.03) | 0.549 |

| Independent Variable | Eating disorder | | | |
| --- | --- | --- | --- | --- |
|  | Model1 | | Model2 | |
|  | OR value (95% CI) | *P* value | OR value (95% CI) | *P* value |
| Age | 1.00 (0.99,1.02) | 0.697 | 1.00 (0.99,1.02) | 0.833 |
| Sex at birth |  |  |  |  |
| Female (Ref = Male) | **2.21 (2.09,2.33)** | **<0.001** | **2.09 (1.98,2.21)** | **<0.001** |
| Residence |  |  |  |  |
| Urban (Ref = Rural) | 1.02 (0.97,1.07) | 0.526 | 1.01 (0.95,1.06) | 0.851 |
| Current annual family income | 1.03 (1.01,1.05) | 0.007 | 1.03 (1.01,1.05) | 0.01 |
| Socioeconomic status | **0.94 (0.93,0.96)** | **<0.001** | **0.97 (0.95,0.98)** | **<0.001** |
| Only child status |  |  |  |  |
| Yes (Ref = No) | 1.04 (0.99,1.09) | 0.144 | 1.05 (0.99,1.10) | 0.097 |
| Ethnicity |  |  |  |  |
| Han (Ref = non-Han Chinese) | 0.93 (0.86,1.00) | 0.063 | 0.94 (0.87,1.01) | 0.108 |
| Smoking |  |  |  |  |
| Yes (Ref = No) | **1.41 (1.32,1.51)** | **<0.001** | **1.41 (1.31,1.51)** | **<0.001** |
| Alcohol Consumption |  |  |  |  |
| Yes (Ref = No) | **1.53 (1.37,1.72)** | **<0.001** | **1.53 (1.36,1.72)** | **<0.001** |
| Exercise | 0.96 (0.93,0.98) | 0.001 | 0.97 (0.94,1.00) | 0.018 |
| Family type (Ref = Nuclear family) |  |  |  |  |
| More than three generation |  |  | **1.13 (1.05,1.20)** | **<0.001** |
| Others |  |  | **1.14 (1.07,1.22)** | **<0.001** |
| Relationship with father |  |  | 1.03 (1.00,1.07) | 0.075 |
| Relationship with mother |  |  | 0.95 (0.92,0.99) | 0.015 |
| Family harmony |  |  | **0.93 (0.91,0.94)** | **<0.001** |
| Education level of mother |  |  | 1.02 (1.00,1.04) | 0.05 |
| Education level of father | = |  | 0.98 (0.96,0.99) | 0.009 |

| Independent Variable | PTSD | | | |
| --- | --- | --- | --- | --- |
|  | Model1 | | Model2 | |
|  | OR value (95% CI) | *P* value | OR value (95% CI) | *P* value |
| Age | 1.00 (0.98,1.01) | 0.643 | 0.99 (0.98,1.01) | 0.449 |
| Sex at birth |  |  |  |  |
| Female (Ref = Male) | **1.43 (1.36,1.52)** | **<0.001** | **1.29 (1.21,1.36)** | **<0.001** |
| Residence |  |  |  |  |
| Urban (Ref = Rural) | 1.05 (1.00,1.11) | 0.075 | 1.01 (0.95,1.07) | 0.745 |
| Current annual family income | 1.02 (1.00,1.04) | 0.066 | 1.01 (0.99,1.03) | 0.352 |
| Socioeconomic status | **0.91 (0.89,0.93)** | **<0.001** | **0.96 (0.94,0.98)** | **<0.001** |
| Only child status |  |  |  |  |
| Yes (Ref = No) | 1.02 (0.97,1.08) | 0.402 | 1.03 (0.97,1.09) | 0.300 |
| Ethnicity |  |  |  |  |
| Han (Ref = non-Han Chinese) | 0.94 (0.87,1.02) | 0.156 | 0.95 (0.88,1.04) | 0.274 |
| Smoking |  |  |  |  |
| Yes (Ref = No) | **1.57 (1.46,1.68)** | **<0.001** | **1.60 (1.49,1.71)** | **<0.001** |
| Alcohol Consumption |  |  |  |  |
| Yes (Ref = No) | **1.658 (1.48,1.86)** | **<0.001** | **1.66 (1.48,1.87)** | **<0.001** |
| Exercise | **0.869 (0.84,0.90)** | **<0.001** | **0.89 (0.87,0.92)** | **<0.001** |
| Family type (Ref = Nuclear family) |  |  |  |  |
| More than three generation |  |  | 1.13 (1.05,1.21) | 0.001 |
| Others |  |  | 1.04 (0.97,1.12) | 0.314 |
| Relationship with father |  |  | **1.11 (1.01,1.15)** | **<0.001** |
| Relationship with mother |  |  | 1.04 (1.00,1.09) | 0.036 |
| Family harmony |  |  | **0.88 (0.87,0.89)** | **<0.001** |
| Education level of mother |  |  | **1.05 (1.03,1.07)** | **<0.001** |
| Education level of father |  |  | 0.98 (0.96,1.00) | 0.073 |

| Independent Variable | Phobia | | | |
| --- | --- | --- | --- | --- |
|  | Model1 | | Model2 | |
|  | *b* (SE) | *P* value | *b* (SE) | *P* value |
| Age | 0.007 (0.018) | 0.698 | 0.002 (0.017) | 0.337 |
| Sex at birth |  |  |  |  |
| Female (Ref = Male) | **1.577 (0.068)** | **<0.001** | **1.212 (0.067)** | **<0.001** |
| Residence |  |  |  |  |
| Urban (Ref = Rural) | -0.219 (0.067) | 0.001 | -0.321 (0.066) | 0.890 |
| Current annual family income | 0.024 (0.025) | 0.345 | -0.009 (0.025) | 0.006 |
| Socioeconomic status | **-0.371 (0.02)** | **<0.001** | **-0.198 (0.020)** | **<0.001** |
| Only child status |  |  |  |  |
| Yes (Ref = No) | **-0.348 (0.066)** | **<0.001** | -0.264 (0.066) | 0.730 |
| Ethnicity |  |  |  |  |
| Han (Ref = non-Han Chinese) | -0.159 (0.101) | 0.114 | -0.129 (0.098) | 0.024 |
| Smoking |  |  |  |  |
| Yes (Ref = No) | **-0.89 (0.087)** | **<0.001** | **-0.845 (0.085)** | **<0.001** |
| Alcohol Consumption |  |  |  |  |
| Yes (Ref = No) | -0.248 (0.151) | 0.101 | **-0.284 (0.146)** | **<0.001** |
| Exercise | **-0.658 (0.035)** | **<0.001** | **-0.545 (0.034)** | **<0.001** |
| Family type (Ref = Nuclear family) |  |  |  |  |
| More than three generation |  |  | 0.071 (0.083) | 0.892 |
| Others |  |  | -0.165 (0.086) | 0.066 |
| Relationship with father |  |  | **0.478 (0.043)** | **<0.001** |
| Relationship with mother |  |  | **0.291 (0.049)** | **<0.001** |
| Family harmony |  |  | **-0.356 (0.016)** | **<0.001** |
| Education level of mother |  |  | **0.086 (0.022)** | **<0.001** |
| Education level of father |  |  | -0.065 (0.023) | 0.219 |

*Note*. *b*: standardized regression coefficient; CI: confidence intervals; CT: childhood trauma; GAD: generalized anxiety disorder; OCD: obsessive-compulsive disorder; OR: odds ratios; PTSD: post-traumatic stress disorder; SAD: social anxiety disorder; Hierarchical logistic regression was performed to find risk factors for six diseases: Depression, GAD, OCD, Autism, Eating disorder, and PTSD according to the cutoff values of the corresponding scales. Since the scale of SAD has no definite cutoff value, hierarchical logistic regression cannot be performed, so hierarchical linear regression is adopted here. This table only presents the results of the second layer of regression. (^*^*P* < .05, ^**^*P* < .01, and ^***^*P* < .001).
