# Supplementary material for: The lasting effects of childhood trauma on developing psychiatric symptoms: A population-based, large-scale comparison study
Source: Glob Ment Health (Camb). 2024 Oct 24;11:e98. doi: 10.1017/gmh.2024.100 (PMC11504938; doi:10.1017/gmh.2024.100)
Supplement: Peer review history

# Editor report: Recommendation: The lasting effects of childhood trauma on developing psychiatric symptoms: A population-based, large-scale comparison study — R0/PR2

**DOI:** 10.1017/gmh.2024.100.pr2

**Affiliations:** Faculty of Business, Technology, and Education, Ilia State University, Georgia

No accompanying comment.

# Editor report: Decision: The lasting effects of childhood trauma on developing psychiatric symptoms: A population-based, large-scale comparison study — R0/PR3

**DOI:** 10.1017/gmh.2024.100.pr3

**Affiliations:** Johns Hopkins University Bloomberg School of Public Health, United States

No accompanying comment.

# Editor report: Recommendation: The lasting effects of childhood trauma on developing psychiatric symptoms: A population-based, large-scale comparison study — R1/PR5

**DOI:** 10.1017/gmh.2024.100.pr5

**Affiliations:** Faculty of Business, Technology, and Education, Ilia State University, Georgia

No accompanying comment.

# Editor report: Decision: The lasting effects of childhood trauma on developing psychiatric symptoms: A population-based, large-scale comparison study — R1/PR6

**DOI:** 10.1017/gmh.2024.100.pr6

**Affiliations:** Johns Hopkins University Bloomberg School of Public Health, United States

No accompanying comment.
